# SANS Spectra with PLUMED: Implementation and Application to Metainference

**DOI:** 10.1021/acs.jcim.3c00724

**Published:** 2023-08-08

**Authors:** Henrique M. Cezar, Michele Cascella

**Affiliations:** Hylleraas Centre for Quantum Molecular Sciences and Department of Chemistry, University of Oslo, PO Box 1033 Blindern, 0315 Oslo, Norway

## Abstract

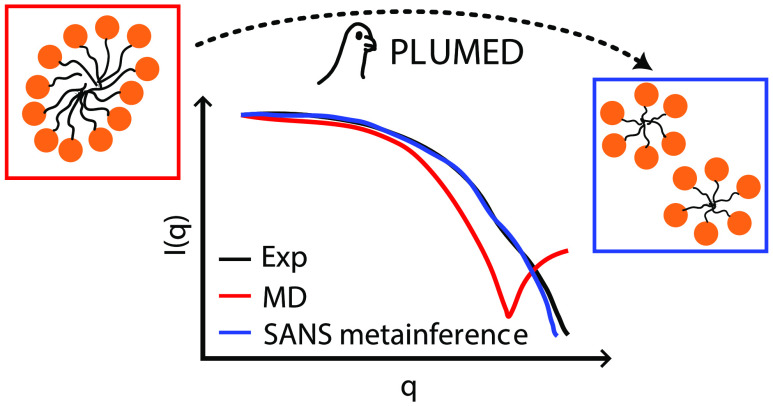

Using small-angle scattering with either X-ray or neutron sources has become common in the investigation of soft-matter systems. These experiments provide information about the coarse shape of the scattered objects, but obtaining more-detailed information can usually only be achieved with the aid of molecular simulations. In this Application Note, we report the implementation of an extension in PLUMED to compute the small-angle neutron scattering (SANS), which can be used for data processing as well for enhanced sampling, in particular with the metainference method to bias simulations and sample structures with a resulting spectrum in agreement with an experimental reference. Our implementation includes a resolution function that can be used to smear the SANS intensities according to beamline error sources and is compatible with both all-atom and coarse-grained simulations. Scripts to aid in the calculation of the scattering lengths when the system is coarse-grained and to aid in preparing the inputs are provided. We illustrate the use of the implementation with metainference by performing coarse-grained simulations of beta-octylglucoside and dodecylphosphocholine micelles in water. With different software and different Hamiltonians, we show that the metainference SANS bias can drive micelles to be split and to change shapes to achieve a better agreement with the experimental reference.

## Introduction

Small-angle scattering (SAS) experiments are a valuable tool in the study of soft-matter and biomolecular systems.^1,2^ Performing small-angle X-ray scattering (SAXS) and small-angle neutron scattering (SANS), the overall shape of objects in solution can be gauged. However, obtaining high-resolution information with these methods is challenging. Molecular modeling can address experimental limitations by accessing the structure with a finer level of detail.^3^ To this end, methods and software to compute and compare SAS spectra from simulations have been proposed over the years.^4−8^ However, simulations are limited by the quality of the potential energy surface used to describe the system; moreover, SAS intensities may not be adequately represented. One possibility is then to reweight the configurations in order to reproduce the spectra.^9,10^ Another possibility is the use of biased simulations.

Using methods such as metainferece,^11^ or other Bayesian,^12^ or maximum entropy methods,^13,14^ one can use the SAS spectra to bias a molecular dynamics (MD) simulation. Therefore, simulations performed with suboptimal force fields can be driven to an improved agreement with the experiment, producing trajectories that span, in principle, the representative ensemble of configurations composing the scattering. For example, using metainference as implemented in PLUMED,^15,16^ the dynamics of polyubiquitin^17^ and the binding of the E2F1/DP1/DNA complex^18^ have been investigated. The current version of the ISDB module of PLUMED,^19^ however, currently performs metainference simulations only with SAXS, either at the atomistic or coarse-grained level.

In this Application Note, we describe the implementation of SANS in PLUMED,^15,16^ including the possibility of metainference simulations using this bias. The SANS intensities are computed using the Debye equation, including resolution effects due to the beam collimation, detector resolution, and wavelength distribution.^20,21^ These resolution effects can be estimated and included in the spectra to smear out the scattering data. Currently, atomistic and user-provided scattering lengths can be used, with the latter being useful for coarse-grained simulations.

We showcase and validate the implementation using it with metainference through coarse-grained MD simulations for beta-octylglucoside (BOG) micelles and dodecylphosphocholine (DPC) micelles in water. These simulations were performed using two different software (GROMACS^22^ and Hylleraas MD^23^), and against two coarse-grained modeling approaches. In the case of BOG, we observe that the MARTINI model^24^ greatly favors the formation of big micelles that do not correspond to the SANS intensities at concentrations close to the critical micelle concentration (cmc). Using metainference with SANS, we can reproduce the correct scattering intensities, with the largest micelle being split. We also tested how metainference with the SANS score can help improve the description of DPC by soft potentials as in the Hamiltonian hybrid particle-field (HhPF)^25^ method. Starting from far from optimal density interaction parameters, we show that metainference can drive the simulations to reproduce the SANS intensities.

## Implementation

### Calculating Scattering Intensities

Following several implementations for computing the SANS intensities, we employ the Debye equation:
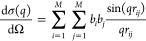
1with dσ(*q*)/dΩ being the coherent scattering cross section, *q* the scattering vector, *b*_*i*_ the scattering length of atom *i*, and *r*_*ij*_ the distance between the atoms *i* and *j* and *M* the total number of scattering centers. These intensities are normalized by dσ(*q*_0_)/dΩ and scaled to match the experimental intensity at *q*_0_, the lowest scattering vector, when experimental data is provided.

If an estimate for the resolution at each *q*, *R*(⟨*q*⟩, *q*), is provided, the intensities are computed as^20,21^
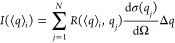
2where ⟨*q*⟩_*i*_ are the *q* values selected by the user, *N* is the number of points around each ⟨*q*⟩_*i*_ used for the integration of the resolution function (typically *N* ≈ 10 is necessary for the convergence), Δ*q* is the integration step, and
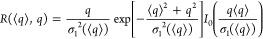
3with *I*_0_ being the zeroth-order Bessel function of the first kind, σ_1_(*q*) the estimate of the resolution at *q*. Formulas to estimate σ_1_(*q*) according to the beamline parameters can be found elsewhere.^21^

### Use with Metainference

Metainference is a Bayesian inference method that incorporates experimental data into molecular simulation.^11^ Here, a bias energy is introduced to the Hamiltonian of the system, considering uncertainties for the error due to a finite number of replicas describing the system, experimental and systematic errors, and errors in the forward model. Using a Gaussian noise model, the energy function (*E*) for *N*_d_ data points and *N*_r_ replicas can be written as^11^
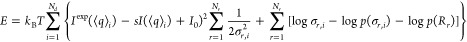
4where *k*_B_ is the Boltzmann constant, *T* the temperature, *I*^exp^(⟨*q*⟩_*i*_) the experimental intensity for the *i*th data point, *s* a scaling factor, *I*_0_ an offset, σ_*r*,*i*_ the effective uncertainty (compromising all sources of error) for the *i*th data point and *r*th replica, *p*(σ_r,*i*_) a prior on the effective uncertainty, and log  *p*(*R*_*r*_) the prior on the structure given by the coordinates *R*_r_ of the *r*th replica. The scaling factor *s* and offset *I*_0_ are optionally sampled with Monte Carlo during the simulation to obtain better agreement with the experimental intensities.

As shown by eq 2, *N* scattering intensities, in principle, at *q* different from those selected by the user, are necessary to obtain a single *I*(⟨*q*⟩_*i*_). For metainference simulations, the gradient of *I*(*q*) is also necessary to compute the biasing forces from eq 4, further increasing the computational cost for the resolution function. We solve this problem by interpolating dσ(*q*)/dΩ using cubic splines. Therefore, dσ(*q*)/dΩ and its derivatives are computed only at ⟨*q*⟩_*i*_ and interpolated to allow the integration over any *q*. *N* evenly distributed *q*_*j*_ values in the interval [⟨*q*⟩_*i*_ – 3σ_1_(⟨*q*⟩_*i*_), ⟨*q*⟩_*i*_ + 3σ_1_(⟨*q*⟩_*i*_)] are selected for the integration. As *R*(⟨*q*⟩, *q*) depends only on *q* and σ_1_(⟨*q*⟩), which are constant during the simulation, we compute it only once, at the beginning of the simulation.

### Calculating Scattering Lengths

**Figure 1:**
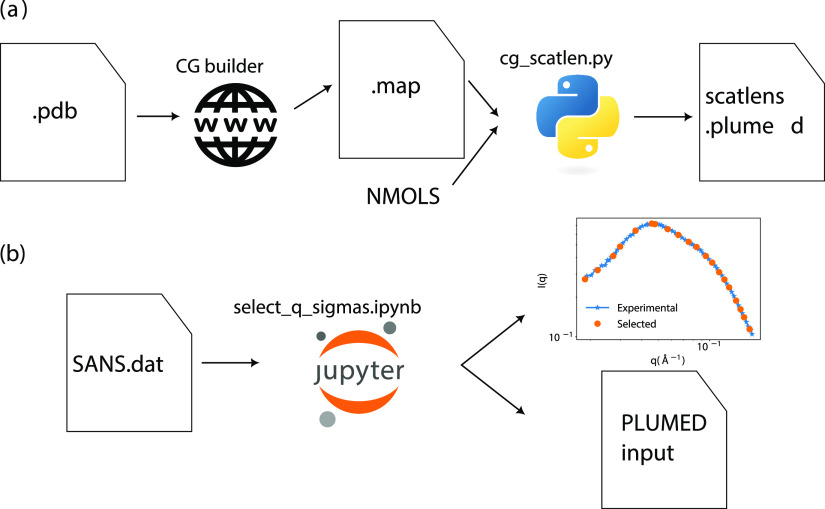
Flowcharts for generating the input files for computing the SANS intensities in the case of a coarse-grained simulation. (a) steps to get the scatlens.plumed file with the scattering lengths for each bead in the system. (b) Steps to select the *q* values from an experimental SANS curve and generate the PLUMED input to compute the SANS intensities or run metainference simulations.

The scattering centers in eq 1 can be either atoms or coarse-grained beads. In the case of atoms, the widely employed scattering lengths of Sears^26^ are used, with the atom types read from the input structure. For coarse-grained beads, mapping of the all-atom structure to the bead must be considered. Similar to previous works,^27,28^ the scattering lengths for each bead are obtained as a sum of the scattering lengths of each atom mapped onto that bead. When preparing the input, it is possible to assign hydrogen atoms as deuterium atoms and vice versa to account for contrast variation experiments. For convenience, we provide a Python script (cg_scatlen.py), which given a mapping from all-atom to coarse-grain (for example, the .map generated by CG Builder^29^), generates the scattering lengths in a format that can be read by PLUMED. For example, generating the scattering lengths based on the map on cgbuilder.map for NMOLS molecules can be achieved with 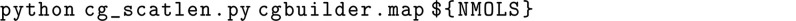 which generates the file scatlens.plumed file that can be read directly in the PLUMED input with the keyword SCATLENFILE inside the SANS action. The content of this file is a sequence of lines containing the keyword SCATLEN with the value for each atom/bead in the system, as shown in the example below for a system with five beads:
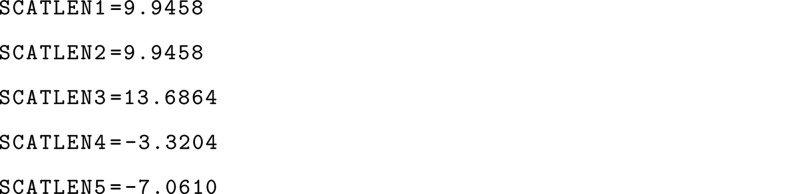


The script has options for building the scattering lengths file for more complex systems, such as –a for appending to the current output file and --start-index to continue from the last used index. It is also possible to set the scattering lengths for each atom and bead directly in the PLUMED input. A workflow showing how to obtain coarse-grain scattering lengths from scratch based on a .pdb file is shown in Figure 1a.

### Preparing SANS Calculation and Metainference Inputs

We use the same syntax for the SANS action as the one used by the SAXS action in PLUMED. The major differences are the introduction of the keywords SCATLENFILE, N, and SIGMARES, which are used to specify a file containing the scattering lengths, the number of points used in the resolution function integration, and the resolution σ_1_ as in eq 3 for a given *q*, respectively. In the example below, we show a PLUMED input to perform a metainference simulation with SANS:

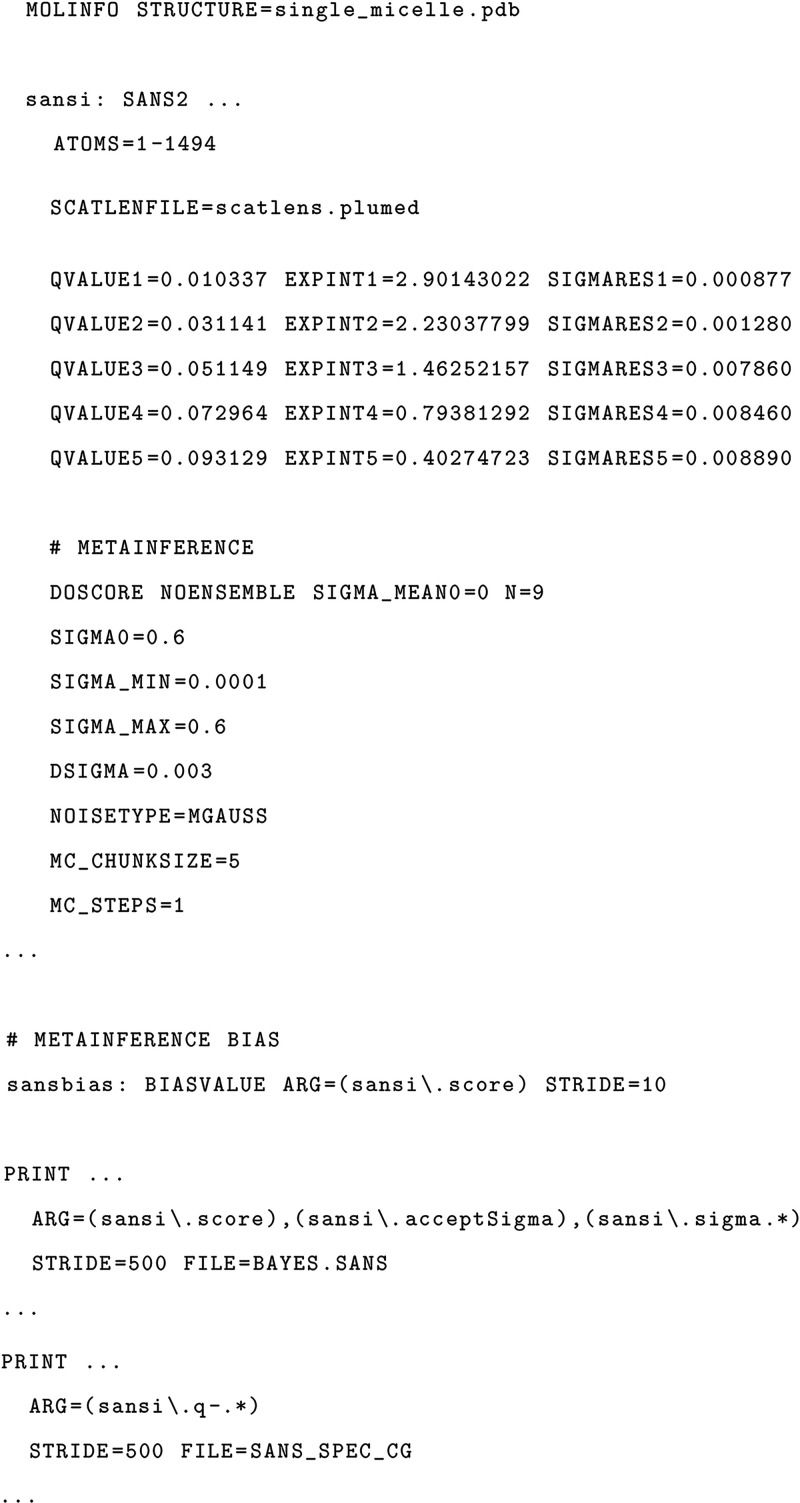



For the ATOMS selection, we consider only the solute atoms to compute the SANS intensities, due to the  scaling of eq 1.

The input file can be obtained with the aid of a provided Python Jupyter Notebook that helps to select the *q* and corresponding experimental intensities and resolution from the user-provided experimental data files. In this Notebook, the user just provides the file containing the experimental curve, initial and final *q*, and desired number of points to obtain the QVALUE/EXPINT/SIGMARES lines of the input and a plot of the selected points over the experimental curve. One can manually select additional points in case some *q* regions (e.g., regions where there is an experimental peak) need to be better described. A workflow for using this Notebook is shown in Figure 1b.

If the user just wants to compute the spectrum from a trajectory, the METAINFERENCE sections of the example input can be disregarded. For metainference simulations, the keywords SIGMA_MIN, SIGMA_MAX, and DSIGMA, corresponding to the range and Monte Carlo sampling of the uncertainty parameters shown in eq 4, should be provided. It is often useful to provide one value for each *q*, instead of one global value for all *q*, in order to have biasing forces that are not too strong or too weak in certain regions of the spectrum. When the forces are too strong, the simulation may be unstable even at small timesteps, while when forces are too weak, the computed SANS spectrum may not converge to the experiment. From our experience, choosing SIGMA_MAX to be ∼8% of the experimental intensity at a given *q*, and choosing SIGMA_MIN and DSIGMA to be ∼25% and 4% of SIGMA_MAX, respectively, gives forces that are appropriately balanced. The Jupyter Notebook can also be used to output these parameters to the PLUMED input file.

## Example Applications

### BOG Micelles

We used the MARTINI maker random builder in CHARMM-GUI^30^ to build a cubic box of side 17 nm with 50 mM of BOG in water. We used GROMACS^22^ to perform simulations at 298.15 K and 1 bar using the CSVR thermostat and SCR barostat. The leapfrog algorithm with a 20 fs time step was used to integrate the equations of motion, and a cutoff of 1.2 nm was used for both Lennard-Jones and short-range Coulomb interactions, and reaction-field for the long-range electrostatic part, with ε_*r*_ = 15. Using MARTINI 2.0^24^ lipidome parameters, we observed the formation of two large micelles, resulting in SANS intensities that do not agree with the experimental spectra and micelles that were unaffected by the metainference bias.

**Figure 2:**
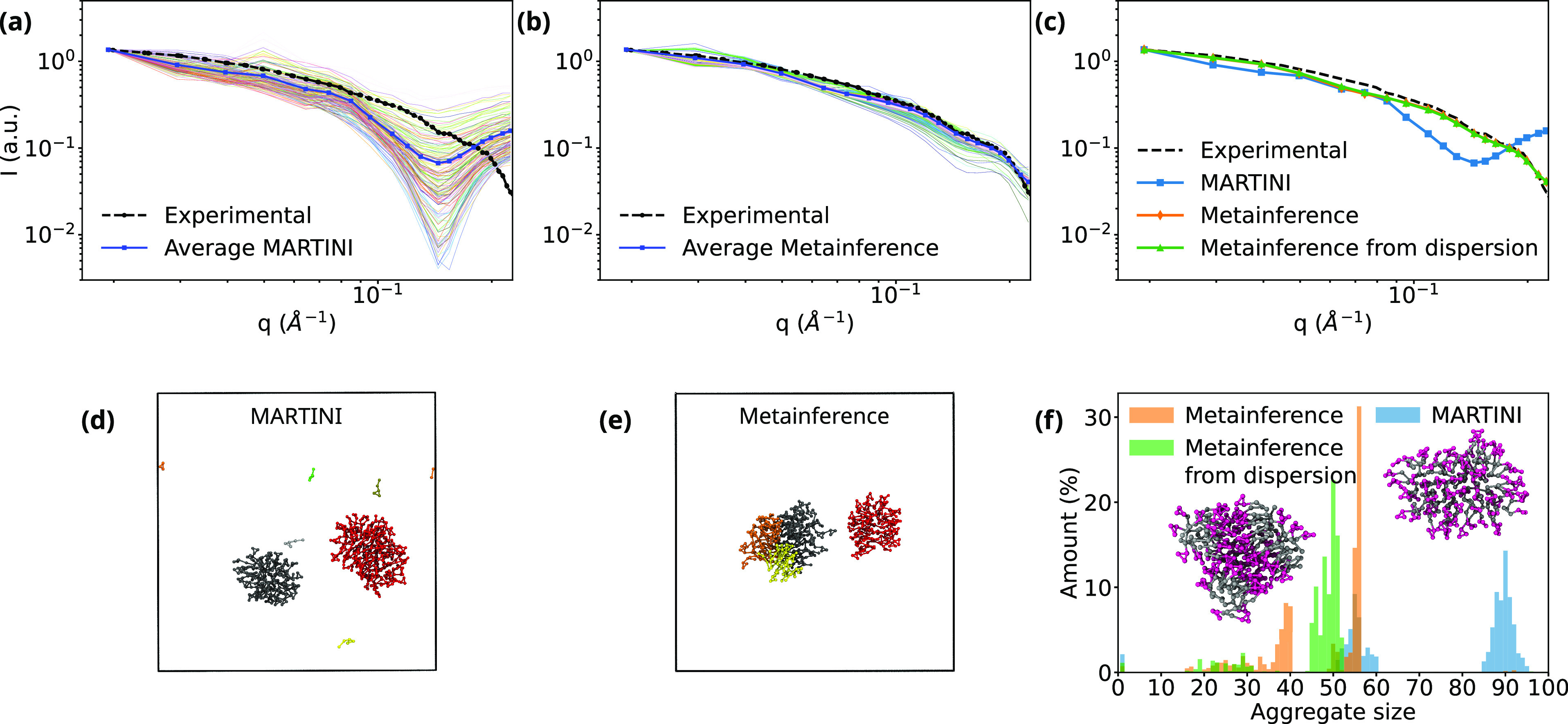
Average SANS intensities computed for MD trajectories using (a) the modified MARTINI 2.0 force field and (b) using metainference for the SANS intensities as described in this work. Colored thin lines represent the scattering of single snapshots. In panel (c), the average curves for the MARTINI and two metainference simulations are plotted with the experimental reference for easier comparison. Snapshots from simulations colored by detected aggregates: (d) unbiased MARTINI simulation and (e) metainference simulation. (f) Aggregate size distribution for the trajectories using the modified MARTINI parameters (blue), metainference biasing toward the SANS intensities starting from the final MARTINI configuration (orange), and metainference starting from a dispersion of BOG in water (green). The experimental curve for the SANS spectrum was obtained from ref (31).

By using more polar beads for glucose (MARTINI P4 type for the two outermost beads and P3 for the bead connected to the alkyl tail) we obtained relatively more loosely bound micelles. The sizes and shapes still did not correspond to the SANS intensities, but they were modified by metainference. Starting from dispersed detergents in water, 3-μs-long unbiased simulations were run, resulting in two micelles with ∼55 and 90 surfactants each. This resulted in SANS intensities that started to increase at ∼0.14 Å^–1^, and deviate from the experimental reference,^31^ as in Figure 2a. The average curve shown in Figure 2a is the result of averaging scattering curves of structures sampled during the whole trajectory.

Metainference simulations were run for 200 ns using a single replica applying the biasing force every 0.1 ps, using the same settings as for unbiased MD. Starting from the final configurations of the unbiased simulations, we observed that the spectrum progressively moved closer to the experimental reference (Figure 2b). Using the software AGGREGATES^32^ to find the clusters, considering the alkyl tail and only the first head bead attached to it, and a maximum distance criterion of 10 Å, we obtain the aggregate size distribution shown in Figure 2f. We observe two aggregates in the case of the unbiased MARTINI simulations, corresponding to micelles with about 55 and 90 surfactants each. However, by metainference, the larger micelle splits into two or three smaller micelles.

A visual inspection of the aggregates (Figures 2d and 2e) shows that, even though the micelles are split, they are not dispersed in the solution. To confirm this, we also ran a metainference simulation for 400 ns starting from randomly dispersed BOG, achieving the same spectrum (Figure 2c). This indicates that the information in the SANS spectrum, at least in this *q* range, does not require the aggregates to be dispersed, and due to the MARTINI trend of having larger aggregates, the smaller aggregates are still glued to each other. As reported in Figure 2f, the size distribution of the aggregates starting metainference simulations from either the MARTINI equilibrium configuration or from dispersion is different. In the first case, simulations are biased by the initial size of the aggregates, with limited surfactant exchange between micelles. Since the SANS spectrum can be reproduced anyway under these conditions, we have more defined peaks, as seen in the orange bars of Figure 2f. Starting from the dispersion, the surfactants are more prone to rearrangement, leading to a more uniform distribution around 50 surfactant molecules, shown in the green bars. We observe, on the other hand, that the bias can split larger aggregates in order to obtain better agreement with the experimental reference regardless of the initial configuration (Figure 2c). Therefore, this is not a limitation of the implementation or method, but a characteristic of the information contained in the SANS spectrum that cannot distinguish between the two distributions.

### DPC Micelles

To test how metainference using the SANS intensities performs in the case of softer potentials, we simulated DPC micelles using the HhPF approach,^25^ as implemented in HylleraasMD (HyMD).^23,33^ We use the same mapping for the surfactant as that used by the MARTINI force field. Simulations were performed in the NVT ensemble at 300 K using the CSVR thermostat, with one DPC micelle composed of 54 surfactants solvated in a cubic water box of ∼8 nm per side. This number of DPC molecules was chosen to match previous theoretical studies^34^ and is within the error of the *n*_agg_ = 58 ± 5 found by our SANS reference data at 10 mM.^35^ An inner time step of 20 fs using the rRESPA integrator was employed, with 5 inner steps for each outer step. Electrostatics is included using the long-range part of the Ewald summation.^33^ A mesh of 64 × 64 × 64 with σ = 0.5 was employed. Since we wanted to test how metainference can be used with the soft HhPF potential, we did not optimize the χ parameters and used the manually assigned parameters, namely (in kJ mol^–1^), χ_NP_ = 4.5, χ_NC_ = 15.0, χ_NW_ = 0.0, χ_PC_ = 8.0, χ_PW_ = 2.0, and χ_CW_ = 35.0. Bonded parameters were taken from the MARTINI^24^ force field. The simulations without metainference were run for 25 ns while the simulations with metainference, starting from those final configurations, were integrated for another 25 ns, applying the bias every 0.5 ps using one single replica. With the accelerated dynamics obtained with HhPF, these lengths were long enough to obtain converged properties. We also performed simulations using MARTINI 2.2^24^ and the same settings as those used for BOG. These simulations were run without metainference, as the SANS intensities were in good agreement with the experiment, consistent with previous results.^36^

**Figure 3:**
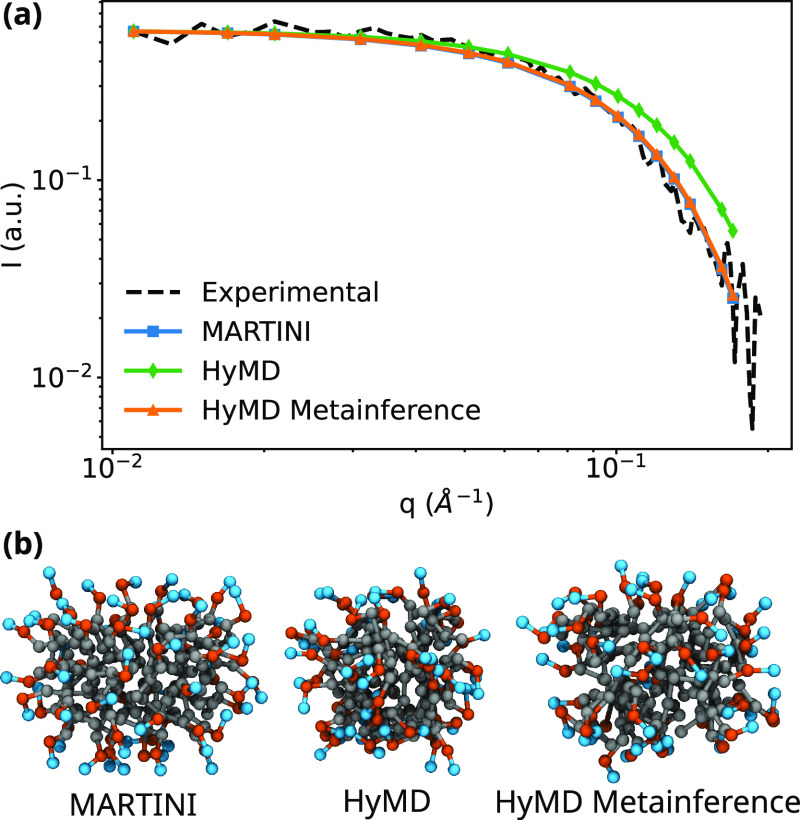
(a) SANS intensities computed for MD trajectories using the MARTINI force field, unbiased HyMD, HyMD using metainference for the SANS intensities as described in this paper, and experimental reference.^35^ (b) Snapshots of the micelles from each simulation, with the amine beads (N) represented in light blue, phosphate beads (P) in orange, and alkyl tail beads (C) in gray.

The average SANS intensities computed over the trajectories for MARTINI, unbiased HyMD, and HyMD with metainference simulations are shown in Figure 3. These averages were computed over at least 100 snapshots for each trajectory. We observed that the provided χ resulted in a SANS spectrum that did not correspond to the experiment. Applying metainference with SANS, we can change the shape of the micelle and reproduce the SANS intensities even with the suboptimal χ parameters.

A summary of some average geometrical properties of the micelles is given in Table 1. We report the radius of gyration (*R*_g_), the three principal moments of inertia (*I*), and also the asphericity parameter (α), which is used to express how spherical the micelles are. This latter parameter is defined as
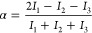
5and α = 0 for a perfectly spherical micelle. For the sake of comparison, we also add the all-atom results of Abel and co-workers.^34^ Overall, we observe that the unbiased HhPF simulations resulted in more compact and spherical micelles with the lowest *R*_g_, α, and moments of inertia across the reported models. The use of metainference with the SANS intensities expands the micelle and makes it more ellipsoidal, making the results obtained with this model closer to the all-atom properties than the MARTINI model.

**Table 1:** Average Shape Parameters for the DPC Micelles

		principal moments of inertia (× 10^–4^ Da nm^2^)			
model	radius of gyration, *R*_g_ (Å)	*I*_1_	*I*_2_	*I*_3_	α
MARTINI	17.4	4.29	3.92	3.31	0.12
HyMD	15.3	3.21	2.98	2.72	0.08
Metainference HyMD	17.2	4.12	3.80	3.37	0.09
AMBER^a^	17.0	4.04	3.72	3.25	0.10
CHARMM^a^	16.8	3.88	3.57	3.21	0.09

a Data from ref (34).

## Conclusions

In this Application Note, we have reported on the implementation and validation of the SANS action in PLUMED. Using the Debye equation and a resolution function that smears the SANS intensities, we successfully compute the SANS spectrum of all-atom and coarse-grained structures. This new implementation also works with metainference, allowing using experimental intensities to bias the simulation toward a better agreement of the SANS intensities. We describe a workflow that can be used to prepare input files for such simulations and provide scripts to aid in this task. To showcase the implementation, we performed coarse-grained simulations of the BOG and DPC micelles in water. The MARTINI force field used to describe the BOG micelles resulted in large micelles with a SANS spectrum that did not match the experimental reference. Employing metainference simulations, we showed that the larger micelles could be split into smaller ones, resulting in a spectrum that matched the experiment. For the DPC micelles, we used the HhPF method to show that even with suboptimal density interaction parameters, we could use metainference to obtain shape parameters in agreement with other coarse-grained and all-atom models. The soft-core repulsion of the HhPF model makes it a good candidate for use with metainference. We expect our implementation to be available in the main PLUMED distribution soon.

## Data Availability

The modified PLUMED version with the implementation of SANS intensities, as described in this work, and the scripts to generate the scattering lengths and the Jupyter Notebook to aid in preparing the input files (selecting *q* from experimental curves and getting SIGMA_MAX, SIGMA_MIN and DSIGMA) are available at the GitHub repository https://github.com/Cascella-Group-UiO/plumed-sans. HylleraasMD (HyMD), the HhPF code used to produce the DPC results contained in this work, is available at the GitHub repository https://github.com/Cascella-Group-UiO/HyMD. All simulation data supporting the findings reported here are shared in https://github.com/Cascella-Group-UiO/publications.
